# Plant Deubiquitinases and Their Role in the Control of Gene Expression Through Modification of Histones

**DOI:** 10.3389/fpls.2017.02274

**Published:** 2018-01-17

**Authors:** Eduardo March, Sara Farrona

**Affiliations:** Plant Developmental Epigenetics Laboratory, Plant & AgriBiosciences Research Centre, National University of Ireland Galway, Galway, Ireland

**Keywords:** deubiquitination, ubiquitin, epigenetics, chromatin, histone modifications, gene expression, Arabidopsis, plant development

## Abstract

Selective degradation of proteins in the cell occurs through ubiquitination, which consists of post-translational deposition of ubiquitin on proteins to target them for degradation by proteases. However, ubiquitination does not only impact on protein stability, but promotes changes in their functions. Whereas the deposition of ubiquitin has been amply studied and discussed, the antagonistic activity, deubiquitination, is just emerging and the full model and players involved in this mechanism are far from being completely understood. Nevertheless, it is the dynamic balance between ubiquitination and deubiquitination that is essential for the development and homeostasis of organisms. In this review, we present a detailed analysis of the members of the deubiquitinase (DUB) superfamily in plants and its division in different clades. We describe current knowledge in the molecular and functional characterisation of DUB proteins, focusing primarily on *Arabidopsis thaliana*. In addition, the striking function of the duality between ubiquitination and deubiquitination in the control of gene expression through the modification of chromatin is discussed and, using the available information of the activities of the DUB superfamily in yeast and animals as scaffold, we propose possible scenarios for the role of these proteins in plants.

## Introduction

Ubiquitination is a post-translational modification that consists of the covalent binding of the small polypeptide ubiquitin to a target protein, either singly or sequentially (polyubiquitination; Akutsu et al., 2016).

Ubiquitination participates in several cell processes such as vesicular trafficking, signalling, subcellular location, regulation of transcription, chromatin structure, and the degradation of a tagged protein (Mukhopadhyay and Riezman, 2007).

Degradation either occurs via the 26S proteasome, a protein complex involved in the degradation of polyubiquitin substrates, while monoubiquitination (ub) or addition of short ub-chains generally targets proteins for degradation via the lysosome (Hicke, 2001; Vierstra, 2009). Ubiquitin-mediated 26S proteasome degradation is a sequential process that starts with the activation of inactive ubiquitin carried out by the E1 (ubiquitin-activating) enzyme in an ATP-dependent manner. The active ubiquitin is transferred from the E1 to the E2 (ubiquitin-conjugating) enzyme that acts as an intermediate and, finally, the E3 (ubiquitin ligase) enzyme mediates the deposition of the active ubiquitin to the target protein, mainly on a lysine residue. Protein ubiquitination can be direct or indirect depending on the E3 that mediates this process (Haas et al., 1982; Ishikura et al., 2010; Zheng and Shabek, 2017).

On the other hand, deubiquitination is carried out by an evolutionary conserved group of proteins known as ubiquitin deconjugating enzymes. Comprising one of the biggest superfamilies, the deubiquitinase superfamily (DUB) counter the action of E3 ligases. The DUBs have three molecular roles: (i) generation of ubiquitin monomers (Chung and Baek, 1999); (ii) regeneration of ubiquitin during the decomposition of ubiquitin-protein conjugates in the 26S proteasome (Amerik and Hochstrasser, 2004); and (iii) deubiquitination of conjugates by releasing intact both the ubiquitin and the target to prevent the degradation of the pre-targeted protein (Taya et al., 1999). This superfamily has five families: ubiquitin-specific proteases (USPs), also called ubiquitin-specific-processing proteases (UBPs) in organisms such as *Arabidopsis thaliana* (Arabidopsis), ubiquitin carboxy-terminal (UCH) proteases, the ovarian tumour proteases (OTUs), the Machado-Joseph disease protein domain proteases or joshephin (MJD) family and the JAB1/MPN^+^/MOV34 (JAMMs) proteases. The first four families are cysteine proteases while the JAMM family are zinc metalloisopeptidases. The DUB family in Arabidopsis contains an estimated 64 members (Yan et al., 2000). However, many of the putative members are still uncharacterised and their molecular activities are still poorly understood.

Ubiquitination/deubiquitination is a highly dynamic process that is ultimately essential for many processes including cell homeostasis, signal transduction, transcriptional gene regulation, protein degradation and endocytosis among others (Hershko and Ciechanover, 1998; Yan et al., 2000; Frappier and Verrijzer, 2011). Here we will summarise the latest plant-based research on this topic, and focus on the role of this dynamic mechanism in the regulation of gene expression.

## The UBP Family: Components and Molecular Activities

Arabidopsis UBP members have redundant functions, but also specific roles in plant development (Liu et al., 2008). This family of UBPs, which possess a highly similar sequence to human USPs proteins, has 27 members in Arabidopsis divided in 14 subfamilies based on specific protein domains (Yan et al., 2000; Zhou et al., 2017). All UBPs in Arabidopsis contain a UBP domain (although these vary in length depending on the protein) and one or more domains that are speculated to be involved in protein–protein interactions (Komander et al., 2009). Among these subfamilies, the cluster composed of UBP10/9/11/5 and 8 all contain DUSP domains, which in humans seem to participate either in protein-protein interactions or in substrate recognition (De Jong et al., 2006). The second, larger, subfamily comprises UBP15/16/17/18 and 19. All these proteins have a Myeloid-type zinc finger domain, Nervy and DEAF-1 (ZnF-MYND) domain, which is found in Eukaryotes such as yeast, Drosophila, mammals as well as plants and might have a role in protein-protein interaction (Gross and McGinnis, 1996). The subfamily formed by UBP12 and 13 have another evolutionary conserved domain called a meprin and TRAF homology (MATH) domain that can act as a folding unit and is sufficient for interaction with receptors (Ye et al., 1999; Sunnerhagen et al., 2002). Another characteristic domain is a zinc finger UBP type (ZnF-UBP) that is shared between the UBP1 and UBP2 subfamily and UBP14, the only member of another subfamily that additionally has an ubiquitin-associated (UBA) domain related with (poly)ubiquitin binding. The ZnF-UBP evolutionary conserved domain has only been reported in some members of the DUB superfamily and maintains functional similarities with other zinc finger domains. In addition, the ubiquitin-like (UBL) domain, which is found in proteins related to the ubiquitin pathway as well as other, non-related, proteins, is found in two subfamilies, the first one composed of UBP6 and UBP7 and the second one represented only by UBP26 (Larsen et al., 1998; Liu et al., 2008). A schematic representation of the members of this family has been previously shown by Liu et al. (2008).

**FIGURE 1:**
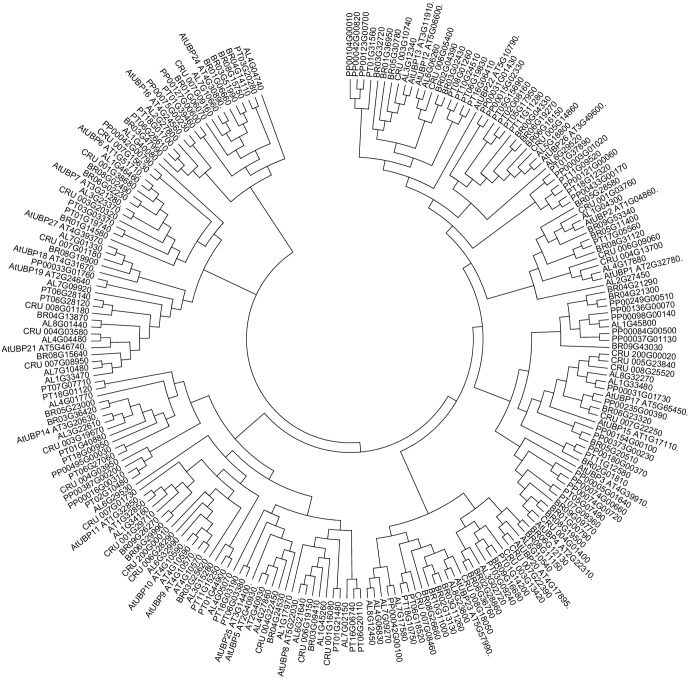
Ubiquitin-specific-processing proteases (UBPs) are highly conserved in plants. *Arabidopsis lyrata* (AL), *Capsella rubella* (CRU*), Brassica rapa* (BRO), *Popolus trichocarpa* (PT), *Physcomitrella patens* (PP). The analysis was conducted in Clustal Omega (Sievers et al., 2014). The tree was edited for easier visualization in MEGA 7. Sequences obtained from PLAZA (Proost et al., 2015).

UBP1 and UBP2 share 62% sequence similarity and also show high sequence conservation with putative members of this subfamily in other plant species (**Figure 1**). UBP2 breaks ubiquitin chains *in vivo* and both UBP1 and UBP2 can also break multi-ubiquitin chains *in vitro*. Analysis of *ubp1* and *ubp2* single mutants producing truncated proteins without functional catalytic domains did not show and altered phenotype when compared to wild-type plants, even under stress conditions. Nevertheless, in the presence of canavanine, an arginine-like compound, the mutant plants display several phenotypic alterations such as shorter roots, chlorotic leaves and reduced growth rate, indicating that UBP1 and UBP2 are involved in the metabolism of this anti-herbivory compound (Yan et al., 2000).

UBP3 and UBP4 localise in the nucleus and are necessary for proper pollen development and transmission (Chandler et al., 1997). Both proteins share redundant function since the single mutants present wild type phenotype, but *ubp3;ubp4* double mutant is not able to mature. Indeed, UBP3 and UBP4 are required for pollen germination because defective sperm production was reported in *ubp3,ubp4* double mutants (Doelling et al., 2007).

UBP6 has a non-canonical Calmodulin motif-binding domain (CaMBD). As calmodulin is involved in the Ca^2+^ signalling pathway as a sensor, this motif in UBP6 suggests the role of Ca^2+^ in protein degradation and/or plant homeostasis. Arabidopsis UBP6 can complement the yeast orthologue *ubp6* mutant background *in vivo.* In addition, the Arabidopsis UBP6 protein allows the recovery of canavanine resistance in the complemented *ubp6* yeast, suggesting a role of the Arabidopsis UBP6 in external stimulus response (Byeong et al., 2005).

UBP12 and UBP13 are involved in plant immunity. Under infection of virulent and avirulent *Pseudomonas syringae* pv. tomato DC3000 (*Pst*DC3000) *UBP12* and *13* genes were found to be upregulated. The study of the single mutants indicates that UBP12 and UBP13 share a redundant function in plant defence response; the *ubp12; ubp13* double mutant shows lethality (Brazma et al., 2003; Ewan et al., 2011). UBP12 and UBP13 have deubiquitinating activity *in vitro*, localise in the cytoplasm as well as in the nucleus and are encoded by ubiquitously expressed genes, which indicates a more general role of these proteins in the plant. This hypothesis was confirmed by analyses of a ubp12-mild;*ubp13* double mutant, which displays an altered flowering time and changes in the circadian rhythms due to misregulation of CONSTANS (CO), a photoperiod pathway regulator, as well as clock genes such as *LATE ELONGATED HYPOCOTYL* (*LHY*), *CIRCADIAN CLOCK ASSOCIATED 1* (*CCA1*), and *TIMING OF CAB EXPRESSION 1* (*TOC1*) (Cui et al., 2013). UBP12 and UBP13 also regulate the ubiquitination of the transcription factor MYC2, a regulator of jasmonate (JA)-mediated responses, which places them as components of the JA signalling pathway (Jeong et al., 2017). Finally, UBP12 and 13 are involved in the process of gene repression that we will discuss in depth in the next chapter. Taken together, the data are consistent with UBP12 and 13 playing key roles in the regulation of plant development and plant defence.

**FIGURE 2:**
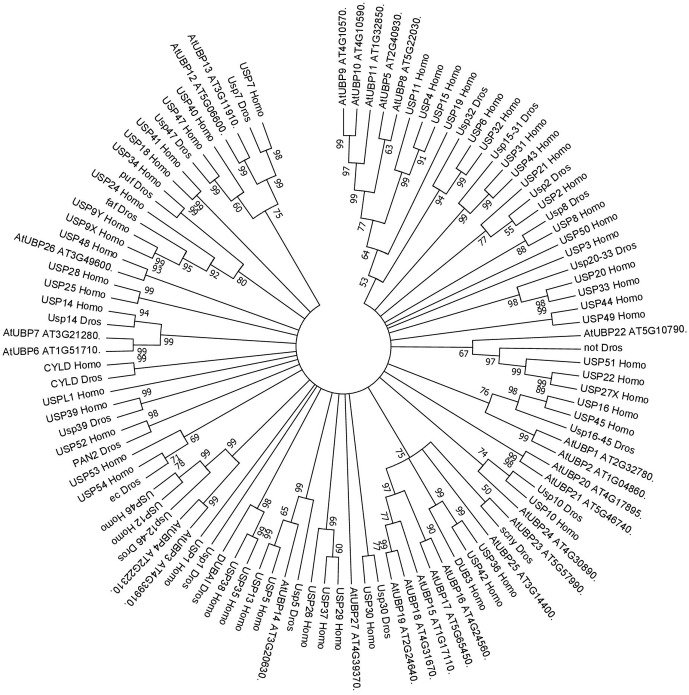
Consensus phylogenetic tree of UBPs/USPs. The tree shows phylogenetic conservation of proteins from Arabidopsis, Drosophila, and humans. The analysis was conducted in MEGA7 (Kumar et al., 2016). The tree was constructed by the Neighbor-Joining method with bootstrap resampling (5000 replicates). The numbers in the nodes indicate bootstrap values. The distances were computed using the Poisson method. Scale bars indicate units of the number of amino acid substitutions per site.

UBP14, which has a highly similar protein sequence to UBP14 in the yeast model organism *Saccharomyces cerevisiae* and USP5 in humans (see **Figure 2**), also seems to have an important role in plant development. This ubiquitin protease has *in vitro* activity against multi-ubiquitin chains and *in vivo* activity against hexameric polyubiquitin. In Arabidopsis, UBP14 is necessary for embryo development, as *ubp14* mutant plants are embryo lethal, displaying an increase in multi-ubiquitin chains as well as ubiquitinated proteins in the seeds resulting in the arrest of embryo development at the globular stage (Doelling et al., 2001). Decrease in *UBP14* expression (*ubp14-*mild*/per1*) affects root development in the absence of phosphate, specifically reducing root hair elongation, suggesting that UBP14 has a role in plant adaptation to different environmental conditions (Li et al., 2010). GFP-UBP14 fusion protein is localised to the nucleus where it is thought to regulate different molecular and developmental process such as endoreduplication and cell and organ growth. Specifically, the UBP domain of UBP14 genetically and physically interacts with UV-B-INSENSITIVE4 (UVA4) to repress anaphase-promoting complex/cyclosome (APC/C) and acts antagonistically with the APC/C activator CELL CYCLE SWITCH52 A1 (CCS52A1), which ultimately represses endoreduplication and so influences cell and organ growth. In addition, *ubp14/da3* mutant presents lower amount of cyclin A2;3 (CDKB1;1) and cyclin-dependent kinase B1;1 (CYCA2;3) that together act as a endoreduplication repressor complex downstream of APC/C (Xu et al., 2016). These results indicate that UBP14 is a determinant for Arabidopsis growth and development through the regulation of the cell cycle.

GFP-UBP15 is localised to the cytosol and nucleus in transient expression experiment in onion cells and, although ubiquitous, its expression is stronger in leaves and flowers where it regulates leaf shape, cell number and flowering time. UBP15 de-ubiquitination activity *in vitro* has also been reported (Liu et al., 2008). Because of its role in controlling cell number, the *ubp15* mutant displays several morphological defects such as shorter roots, smaller flowers and shorter fruits. These results suggests a role of UBP15 in plant size as well as leaf development. The activity of UBP15 seems to be partially redundant to UBP16 despite having higher sequence similarity to UBP17 (see Liu et al., 2008). In addition to cell number, UBP15 also affects seed size promoting cell proliferation in the integuments of ovules. UBP15 overexpression lines develop bigger seeds compared to wild type, while the *ubp15* mutants form smaller seeds, again highlighting its role in plant development (Du et al., 2014).

On the other hand, UBP16 has a role in tolerance to saline abiotic stress conditions by regulating the plasma membrane Na^+^/H^+^ antiport activity and promoting the accumulation of K^+^ instead of Na^+^. *ubp16* mutant displays hypersensitivity to salt stress and accumulate more sodium and less potassium. Besides, UBP16 stabilises SERINE HYDROXYMETHYLTRANSFERASE1 (SHM1), reported as a salt tolerance regulator, and also directly affects the Na^+^/H^+^ antiport/K^+^ activity. In addition, UBP16 regulates cell death via the SHM1-mediated reactive oxygen species inhibitor pathway (Zhou et al., 2012). Another member of this subfamily, UBP19, may have an essential role in plant development as *ubp19* mutants are embryo lethal (Liu et al., 2008).

Abscisic acid signalling and salt and drought tolerance is mediated by the USP domain of UBP24, which affects stomata closure. In addition, UBP24 can form homodimers and cleave UBQ1 into ubiquitin monomers *in vitro* (Zhao et al., 2016).

The activities of UBP26 have been related to transcriptional control through deubiquitination of specific histone tails that will be discussed below.

Finally, UBP27 seems to be involved in mitochondria morphogenesis. UBP27 localises to the mitochondria and cleaves polyubiquitin *in vitro*. Although the *ubp27* mutant does not show any phenotypic variation even in the mitochondria, UBP27 overexpression lines display changes in mitochondria shape (Pan et al., 2014).

## UCH Family: Components and Molecular Activities

**FIGURE 3:**
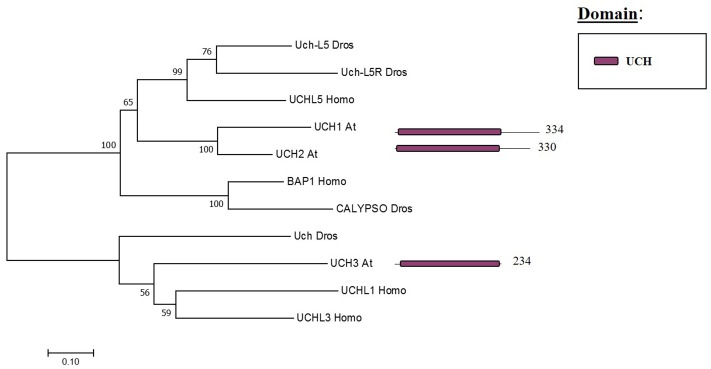
Arabidopsis UCH family and the high sequence similarity proteins in Humans and Drosophila. Phylogenetic analysis showing the 3 members of the UCH family based on protein sequence percentage of similarity and their domains. The analysis was conducted in MEGA7 (Kumar et al., 2016). The tree was constructed by the Neighbor-Joining method with bootstrap resampling (5000 replicates). The numbers in the nodes indicate bootstrap values. The distances were computed using the Poisson method. Scale bars indicate units of the number of amino acid substitutions per site. The numbers in the domain representation indicate the amino acids number.

The Arabidopsis UCH family has only three members (UCH1, 2, 3). All three share an N-terminal as well as the catalytic domain; nevertheless, only UCH1 and UCH2 show a high similarity of the C-terminal sequence (see **Figure 3**). In addition, the three members have a C-terminal extension, which in humans was reported to be an active-site crossover loop that is necessary for substrate size specificity, blocking the catalysis of multimeric ubiquitin (Popp et al., 2009).

UCH2 can release ubiquitin *in vitro* from the targets linked as peptides as well as isopeptides. UCH1/2 have a strong impact on shoot development, producing changes in the architecture of the inflorescence. The analysis of the *uch1-1;uch2-1* double mutant showed an enhancement of AUX/IAA protein turn-over, affecting the shoot architecture due to changes in auxin signalling patterns. The opposite effect was observed using the UCH1 overexpression line. Both *UCH1/2* are expressed ubiquitously, although *UCH2* expression is higher than *UCH1*. UCH1 and 2 localise in the cytoplasm as well as in the nucleus (Yang et al., 2007). Strikingly, in contrast to the role of these proteins in yeast and humans, UCH1/2 does not seem to form part of the Arabidopsis 26S proteasome, although a truncated version of the protein was able to interact with subunits of the 26S proteasome *in vivo*. In addition, UCH1/2 interact with each other *in vivo* forming heterodimers in addition to subunits of the transcription-export complex 2 (TREX-2), which mediates the export of mature mRNA and tagged genes that are being transcribed (Tian et al., 2012). Future research will be required to elucidate the activities of the third member of the family in Arabidopsis and other plant species.

## OTU Family: Components and Molecular Activities

**FIGURE 4:**
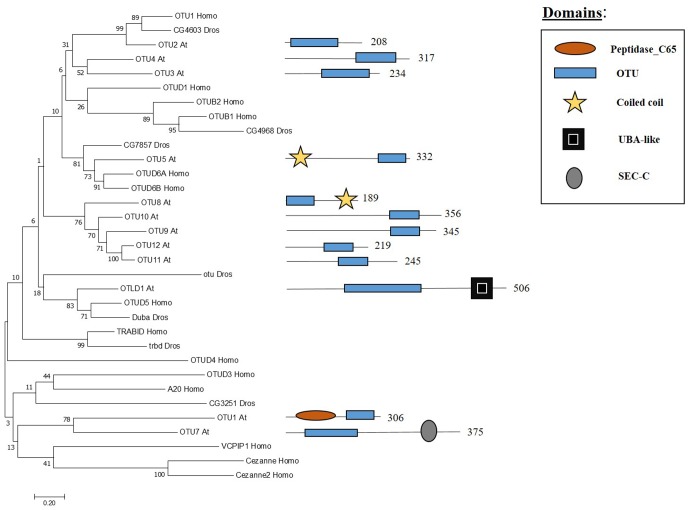
Arabidopsis OTU family and the high sequence similarity proteins in Humans and Drosophila. Phylogenetic analysis showing the 12 members of the OUT family based on protein sequence percentage of similarity and their domains. The analysis was conducted in MEGA7 (Kumar et al., 2016). The tree was constructed by the Neighbor-Joining method with bootstrap resampling (5000 replicates). The numbers in the nodes indicate bootstrap values. The distances were computed using the Poisson method. Scale bars indicate units of the number of amino acid substitutions per site. The numbers in the domain representation indicate the amino acids number.

The OTU domain, that gives its name to this family of proteases, was first described in Drosophila through studies focused on the *ovarian tumour (otu)* gene (Steinhauer et al., 1989) and later on the domain was recognised in proteins of eukaryotes, viruses and bacteria (Makarova et al., 2000). In Arabidopsis 12 genes have been found to encode OTU domain proteins, which share similarity with other OTU proteases in metazoans (**Figure 4**). Some Arabidopsis OTU proteins have isoforms with a disrupted OTU domain due to alternative splicing. In addition to the OTU domain, with its characteristic catalytic Cys-His-Asp/Asn triad, Arabidopsis OTUs proteases can also have other domains, such as a UBA-like domain in OTUBAIN-LIKE DEUBIQUITINASE 1 (OTLD1) isoforms, a coiled-coil domain in OTU5 isoforms and a putative nucleic acid binding SEC-C motif in OTU7a. In addition the OTU domain displayed differences both with substrate and specificity. OTU1/2/3/4c/7a/9/10 cleave lysine-48 (K-48) linked ub chains *in vitro*, suggesting a role in the proteasome degradation pathway, and can also hydrolyse lysine-63 (K-63) linked ub chains, involved in other biological processes such as endocytosis, DNA repair and complex assembly. OTU1 also cleaves ub linear dimers, trimers and tetramers; whereas, OTU3/4c has only been shown to hydrolyze trimers and tetramers (Radjacommare et al., 2014).

The only OTU protease described at the molecular level in Arabidopsis so far is OTLD1. Using OTLD1 overexpression lines, different defects in Arabidopsis were reported such as increased number of flowers per inflorescence and enhanced plant growth due to the increase of cell size. OTLD1 represses genes involved in plant growth, cell expansion, hormone signalling and transition from cell division to cell expansion such us *WUS*, *OSR2* or *ABI5* among others through H2Bub1 deubiquitination at these *loci*. In addition, a decrease of H3K4me3 was also reported in these genes (Keren and Citovsky, 2016). These results suggest that OTU domain proteases may have different roles and further research will be needed in the characterisation of these proteins.

## MJD Family: Components and Molecular Activities

**FIGURE 5:**
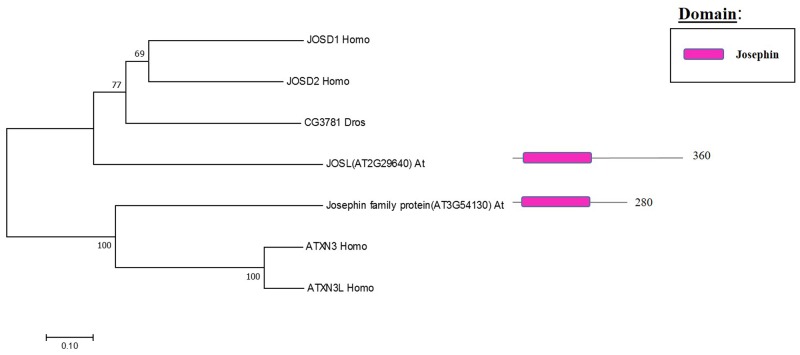
Arabidopsis Josephin family and the high sequence similarity proteins in Humans and Drosophila. Phylogenetic analysis showing the 2 members of the Josephin family based on protein sequence percentage of similarity and their domains. The analysis was conducted in MEGA7 (Kumar et al., 2016). The tree was constructed by the Neighbor-Joining method with bootstrap resampling (5000 replicates). The numbers in the nodes indicate bootstrap values. The distances were computed using the Poisson method. Scale bars indicate units of the number of amino acid substitutions per site. The numbers in the domain representation indicate the amino acids number.

The Machado Joseph disease protein domain (MJD) proteases have not been described in Arabidopsis. This family has four members in humans and evolutionary analysis demonstrates the presence of at least two genes with high sequence similarity to the human MJD protease Ataxin-3 (ATXN3) in Arabidopsis, which acts as a histone-binding protein that mediates gene repression interacting with key regulators of transcription (**Figure 5**) (Li et al., 2002). Therefore, considering this data from humans, Arabidopsis MJD proteins may be involved in the epigenetic regulation of gene expression, an exciting hypothesis that will need further characterisation.

## JAMM Family: Components and Molecular Activities

**FIGURE 6:**
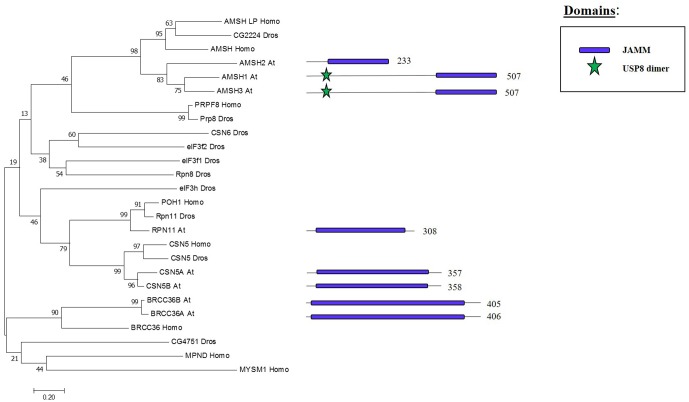
Arabidopsis JAMM family and the high sequence similarity proteins in Humans and Drosophila. Phylogenetic analysis showing the 8 members of the JAMM family based on protein sequence percentage of similarity and their domains. The analysis was conducted in MEGA7 (Kumar et al., 2016). The tree was constructed by the Neighbor-Joining method with bootstrap resampling (5000 replicates). The numbers in the nodes indicate bootstrap values. The distances were computed using the Poisson method. Scale bars indicate units of the number of amino acid substitutions per site. The numbers in the domain representation indicate the amino acids number.

Whereas the previous three families are cysteine proteases, the JAMMs are zinc metalloproteases in which their characteristic MPN+ domain have two zinc ions. One of the two zinc ions activates a water molecule to promote the cleavage of the isopeptide bond on a K-63 linked ub chains (Maytal-Kivity et al., 2002; Sato et al., 2008). The phylogenetic tree in **Figure 6** shows the eight JAMM proteases of Arabidopsis.

ASSOCIATED MOLECULE WITH THE SH3 DOMAIN OF STAM3 (AMSH3), one of the three Arabidopsis proteins that show high similarity to human JAMM protease AMSH, cleaves K-48 and K-63 linked ub chain *in vitro*. The *amsh3* mutant shows lethality at seedling stage and this was related to the proper vacuolar biogenesis at late embryogenesis stage. The lack of AMSH3 impairs the transport from the plasma membrane to the vacuole that is going to ultimately affect the ubiquitin-dependent endocytosis (Isono et al., 2010). AMHS3 also interacts with ENDOSOMAL COMPLEX REQUIRED FOR TRANSPORT-III (ESCRT-III) subunits VPS2.1 and VPS24.1, both involved in intracellular trafficking, through its Microtubule Interacting and Transport (MIT) domain, which is essential for AMSH3 function *in vivo* (Katsiarimpa et al., 2011, 2014). AMSH1 also interacts with the ESCRT-III subunit VPS2.1 which is important for the proper autophagic degradation process (Katsiarimpa et al., 2013).

BRCA2-CONTAINING COMPLEX 36 HOMOLOG A (BRCC36A) and BRCC36B are two other JAMM proteases in Arabidopsis, which display high sequence similarity to the mammalian BRCC36 that is involved in the DNA damage response pathway as subunit of the BRCA1-A complex, one of the most characterised complex involved in tumour suppression in humans (Her et al., 2016). BRCC36A/B are expressed ubiquitously and neither the single mutants nor the *brcc36a/b* double mutant shows any developmental problems. On the other hand, *brcc36a* presents defects in the intra- and inter-chromosomal homologous recombination, BRRC36A localises in the nucleus after genotoxic stress and is involved in the DNA crosslink repair in somatic cells showing an epistatic effect to BRCA1 in this process (Block-Schmidt et al., 2011). These results suggest that BRCC36A/B could affect different complexes, as it has been shown in other organisms, to regulate different molecular processes.

Two paralogous genes, *CSN5A* and *CSN5B*, encode CSN5 JAMM protease isoforms in Arabidopsis (Kwok et al., 1998). CSN5 is the core subunit of the COP9 signalosome (CSN), a conserved complex involved in signalling as well as development, and promotes protein degradation through removal of the Related to Ubiquitin (RUB) [also known as neddylin (NEDD8)] protein (Cope et al., 2002). *csn5b* single mutants do not show phenotypic defects, *csn5a* mutant has growth defects and *cns5a/b* double mutants are lethal (Dohmann et al., 2005). *CSN5A* expression is higher compared to *CSN5B* in Arabidopsis, both CSN5A and CSN5B are the core subunit of the CSN complex. Furthermore, CSN5A/B may have different functions since VTC1, an enzyme involved in the synthesis of vitamin C, interacts with CSN5B but not with CSN5A. (Gusmaroli et al., 2004; Wang et al., 2013). These results suggest that CSN5A/B have only partial redundant activities and, therefore, further investigation will be required to fully decipher their combined and exclusive roles.

RPN11 was found in the purification of the proteasome complex in yeast (Glickman et al., 1998). RPN11 is a subunit of the 26S yeast proteasome lid subcomplex involved in the deubiquitination of proteasome substrates when the proteins arrive for their degradation (Verma, 2002). RPN11 activity is supported by RPN8. These two proteins form a heterodimer which alters the RPN11 MNP^+^ domain to allow RPN11-mediated cleavage of ubiquitin from different tagged substrates in a non-specific manner (Worden et al., 2014). In Arabidopsis, a RPN11 homolog was co-purified with the 26S proteasome, specifically as part of the 19S Regulatory Particle (Book et al., 2010).

Further research is necessary in the characterisation of this family and, considering that several JAMM proteases work as subunits in complexes that are not fully described in plants, a deeper knowledge of their molecular activities will be essential to elucidate the role of these complexes.

## DUBs Influence on Gene Expression Regulation: H2A and H2B

A crosstalk between DUBs and the regulation of gene expression by epigenetic changes has been described in humans, Drosophila and yeast and, based on the evolutionary conservation of the superfamily (**Figures 1**, **2**), future advances of this exciting topic in plants may be expected. Indeed, novel information on the role of different Arabidopsis DUBs in the epigenetic regulation of gene expression is emerging.

Epigenetics studies the mechanisms that produce stable and heritable changes in gene expression patterns without changes in the DNA sequence (Wolffe and Matzke, 1999). Different epigenetic processes have been observed acting at several levels, such as chromatin remodelling, modifications in non-coding RNAs, histones variants, DNA methylation and post-translational histone tail modifications (Allis and Jenuwein, 2016).

**FIGURE 7:**
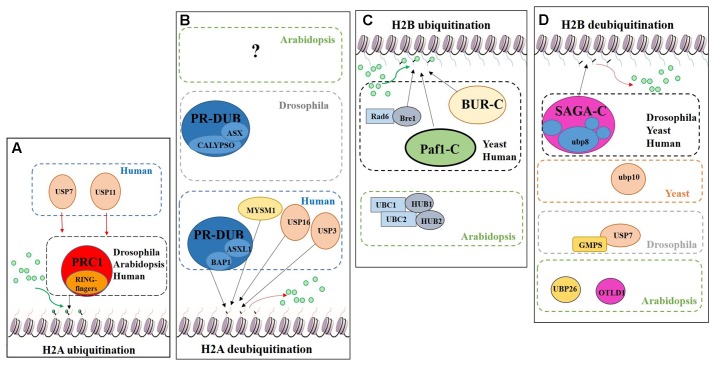
Schematic model showing the regulation of DUBs and support complexes involved in H2A and H2B monoubiquitination/deubiquitination. The figure represents four processes, H2A monoubiquitination, H2A deubiquitination, H2B monoubiquitination and H2B deubiquitination. Ubiquitin is represented by green circles. **(A)** H2A monoubiquitination. PRC1 function is conserved in Eukaryotes. In humans two additional USPs have been described in this process. **(B)** H2A deubiquitination. The role of PR-DUB has been describe in humans and flies, as well as three additional DUBs. The proteins involved in this process in Arabidopsis are still unveiled. **(C)** H2B monoubiquitination. Bre1 and two complexes (BUR-C and Paf1-C) are involved in the deposition of ubiquitin on the H2B. In Arabidopsis only the orthologues of Bre1 and his partner Rad6, have been described in relation to H2Bub1. **(D)** H2B deubiquitination. Several DUBs are involved in this process in different organisms. Arabidopsis has a SAGA-like complex but its role in H2B deubiquitination has not been demonstrated.

Regarding histone tail modifications, more than 30 different epigenetic marks have an impact on the structure of the chromatin, by increasing or decreasing its level of compaction and, subsequently, preventing or promoting gene transcription (Berger, 2007; Pfluger and Wagner, 2007). The deposition of these marks is mediated by chromatin remodelling complexes (Rando and Ahmad, 2007). One of these post-translational histone tail modifications is ubiquitination, which can be deposited on the tails of H2A and/or H2B causing opposite transcriptional results (**Figure 7**).

### H2A

The Polycomb group (PcG) proteins are a highly evolutionary conserved group involved in the control of gene expression modulating specific repressive epigenetics marks at histone tail level, including ubiquitination (Chittock et al., 2017). Three major complexes have been distinguished in metazoans: Polycomb Repressive Complex 1 (PRC1), Polycomb Repressive Complex 2 (PRC2) and Polycomb Repressive DeUBiquitinase (PR-DUB) complex (Müller et al., 2002; Grimaud et al., 2006; Li et al., 2007; Jamieson et al., 2013). PRC1 mediates gene repression *via* catalysis of H2A monoubiquitination (H2Aub1) (Wang et al., 2004; Calonje, 2014) (**Figure 7A**). In mammals, the PRC1 subunits Bmi1, Ring1A and Ring1B have E3 ubiquitin ligase activity, regulating H2Aub1 deposition (Cao et al., 2005). PRC2 mediates the deposition of trimethylation of histone 3 on lysine 27 (H3K27me3), a repressive epigenetic mark (Schatlowski et al., 2010; Mozgova et al., 2015). Recent published data show the *in vivo* interaction between UBP12 and 13 with LIKE HETEROCHROMATIN PROTEIN 1 (LHP1), a PcG related protein that connects PRC1 and PRC2 because acts as a reader of H3K27me3 and physically interacts with members of both PRCs, also repressing some PcG targets (Derkacheva et al., 2016).

H2A monoubiquitination (H2Aub1) is related to transcriptional repression. This mark is deposited on the H2AK121 in Arabidopsis, H2AK119 in humans and H2AK118 in Drosophila but is absent in yeast. In Arabidopsis, PRC1 RING-finger orthologues were identified, specifically three Bmi1 proteins (AtBMI1A, AtBMI1B and AtBMI1C) and two Ring1 proteins (AtRING1a and AtRING1b) (Sanchez-Pulido et al., 2008). These five PRC1 RING-fingers can monoubiquitinate the H2A.1 isoform *in vitro* and AtBMI1A/1B are necessary for H2Aub1 *in vivo* due to the decrease of H2Aub1 in *AtBMI1A/1B* double mutant background. However, *in vivo* monoubiquitination of H2A by AtRING1a/1b has yet to be demonstrated (Bratzel et al., 2010).

H2A deubiquitination is usually associated with transcriptional activation (Nakagawa et al., 2008). In mammals, MYSM1, a member of the JAMM DUB family was the first enzyme reported to affect H2Aub1 levels (**Figures 6**, **7B**). MYSM1 overexpression shows a decreased level of H2Aub1 and the *mysm1* mutant in human embryonic kidney cell lines (HEK293T) displays an accumulation of H2Aub1. Changes in the levels of H2Aub1 also alter the enrichment of other epigenetic marks. For instance, H2A deubiquitination correlates with an increase of H1 phosphorylation, which is related with gene activation (Zhu et al., 2007). The phylogenetic analyses of the plant JAMM family did not allow the identification of a MYSM1-like protein (**Figure 6**) and, thus, molecular data will be required to demonstrate whether a plant JAMM protein might develop a similar activity.

In HeLa cells, USP16 deubiquitinates H2A *in vitro* and *in vivo* and USP16 knock-down RNA line shows accumulation of H2Aub1, which affects the cell growth ratio and regulates the expression of a HOX gene*, HOXD10* (Joo et al., 2007). The closer proteins in Arabidopsis based on high sequence similarity are UBP1 and UBP2 (**Figure 2**). Nevertheless, the role of these two proteins in H2A deubiquitination have not been characterised.

USP3 also affects the cell cycle progression and at a molecular level mediates the deubiquitination of H2Aub1 and γ-H2AX under DNA damage response (Nicassio et al., 2007; Sharma et al., 2014). Based on our phylogenetic analysis, we do not detect a clear high similarity candidate in Arabidopsis’s UBPs.

The PR-DUB complex counteracts PRC1-mediated repression, as it removes H2Aub1 (Scheuermann et al., 2012) (**Figure 7B**). In contrast to PRC1 and PRC2, a functional PR-DUB has not been described in Arabidopsis or any other plant.

The UCH family has an important role in the levels of the H2Aub1 mark. The UCH protein CALYPSO is the catalytic subunit of the PR-DUB of Drosophila (**Figure 7B**). PR-DUB mutants display an accumulation of H2Aub1 and misregulation of homeotic genes, indicating the essential role of this complex in the dynamics of H2Aub1 (Alonso et al., 2007; Scheuermann et al., 2010). The CALYPSO orthologue in humans is the tumour suppressor BRCA-1-associated protein 1 (BAP1) (Sahtoe et al., 2016). Strikingly, the ubiquitination and deubiquitination at H2A is a highly dynamic process in crosstalk with other PRCs as well as with other epigenetic marks. In Arabidopsis there are no clear orthologues of CALYPSO (see **Figure 3**) and complexes involved in H2A deubiquitination have indeed not been reported. However, the conservation of the H2Aub1 mark suggests a similar dynamic control by still unidentified DUB proteins or DUB protein complexes. Considering the UCH domain is conserved in the three Arabidopsis’s UCH members, the regulatory role of BAP1/CALYPSO might have been acquired by one of these proteases in Arabidopsis.

### H2B

While H2Aub1 is a repressive transcriptional mark, H2B monoubiquitination (H2Bub1) generally plays a role in transcriptional activation. While in Arabidopsis this mark is deposited on the K143 of the H2B tail, in other organisms H2Bub1 occurs on different lysine residues of the H2B. Similar to the situation in mammals and yeasts, genome-wide distribution of H2Bub1 in Arabidopsis correlates with other active epigenetic marks, such as H3K4me3 and H3K36me3 (Roudier et al., 2011).

Radiation sensitivity protein 6 (Rad6) was the first yeast protein reported to have E2 activity in the deposition of the H2Bub1 *in vitro* and *in vivo* (Robzyk et al., 2000) (**Figure 7C**). Rad6 co-operates with the E3 enzyme Bre1, which is essential for H2Bub1 *in vivo*. In the monoubiquitination of H2B several support complexes are needed. The Paf1 complex is necessary for proper H2Bub1 since defective single mutants of components of this complex showed a loss of H2Bub1 (Wood et al., 2003) (**Figure 7C**). In humans these factors and their functions are conserved (Kim et al., 2005; Zhu et al., 2005). The activity of the BUR complex is also required since the defective mutant of Bur2, one of the complex components, shows a decrease in H2Bub1 (Wood et al., 2005) (**Figure 7C**). In Arabidopsis, both, Rad6 (UBC1, UBC2 and UBC3) and Bre1 (HUB1 and HUB2) play a repressive role in the control of flowering time through the activation of *FLOWERING LOCUS C* (*FLC*), the main repressor of flowering in Arabidopsis, by the deposition of ubiquitin from UBC1/2 to H2B guided by the E3 HUB1/2 (Xu et al., 2009). Subsequently, H2Bub1 enrichment in *FLC* promotes the deposition of H3K4me3 and H3K36me3 (Cao et al., 2008).

Considering the role of H2Bub1, it is obvious that removal of this mark entails a reduction of transcription. In yeast, Ubp8 and Ubp10 deubiquitinate H2Bub1. Ubp10 acts independently, while Ubp8 is a subunit of the Spt-Ada-Gcn5-acetyltransferase (SAGA) complex (Gardner et al., 2005) (**Figure 7D**). Orthologues of the yeast Ubp8 exist in Drosophila (Non-stop) and humans (USP22), as well as other SAGA complex subunits (Zhang et al., 2008; Weake et al., 2008). The Arabidopsis SAGA complex has a highly similar UBP protease (UBP22), but no H2B deubiquitination activity has yet been reported for either UBP22 or the SAGA complex. On the other hand, the role of SAGA in the control of gene expression through histone acetylation is conserved (Moraga and Aquea, 2015; Kim et al., 2015) and, hence, conservation of its involvement in H2B deubiquitination is a plausible hypothesis. In Drosophila, USP7 contributes to homeotic gene silencing guided by Polycomb (Pc). *In vitro* analyses showed that USP7 interacts with guanosine 5-monophosphate synthetase (GMPS) and that this interaction is required for USP7 H2B deubiquitinase activity (**Figure 7D**) (Van Der Knaap et al., 2005).

In humans, USP7 and USP11 physically interact with members of PRC1 *in vivo*, such as Mel18, Bmi1 and Ring1. USP7 deubiquitinates H2A and H2B *in vitro* and changes in Bmi1 and Ring1 ubiquitin levels were reported in *USP7* and *USP11* overexpression lines. *usp7* and *usp11* mutants in human fibroblasts result in de-repression and loss of PRC1 binding to the tumour suppressor *INK4α* locus (Maertens et al., 2010). These results suggest that USP7 and USP11 have a double role in PRC1 functions: (i) regulating the activity of PRC1 through modification of the ubiquitin levels of the catalytic subunits: (ii) acting on PcG targets as direct partners of PRC1. In Arabidopsis, UBP12 and UBP13, which share a similar protein sequence to USP7 (see **Figure 2**), interact *in vivo* with LHP1, a PcG protein. It has been shown that UBP12 binds to PcG targeted chromatin and is needed for the deposition of H3K27me3 in PcG target genes (Derkacheva et al., 2016). Finally, Derkacheva and colleagues showed how UBP12 and UBP13 also contribute to gene silencing in heterochromatin, although its role is smaller compared to UBP26 in this process, sharing this function with the Drosophila USP7.

UBP26 and OTLD1 are the only DUBs described with H2Bub1 deubiquitination activity in Arabidopsis (**Figure 7D**). *toc1* was identified as a suppressor of mutations affecting *REPRESSOR OF SILENCING1 (ROS1)*, a DNA demethylase involved in suppressing gene silencing (Sridhar et al., 2007). *ubp26* shows higher levels of H2B monoubiquitination (H2Bub1) as well as decreased non-CpG DNA methylation. These results indicate that UBP26 does indeed deubiquitinate H2B (**Figure 7D**) and furthermore that this post-translational modification is required for the deposition of the repressive mark H3K9me2, which in turn is needed for gene silencing through DNA methylation in heterochromatin. Mutations in *UBP26* arrest embryo development, similar to some PcG members mutants, upregulating the expression of *PHERES1 (PHE1)* due to low enrichment of H3K27me3 at the *PHE1* locus (Luo et al., 2008). UBP26 affects flowering time, mediating in the silencing of *FLC* expression due to H2Bub1 deubiquitination on *FLC* chromatin. As a consequence, *ubp26* mutant displays an early flowering phenotype as well as higher global level of H2Bub1 (Schmitz et al., 2009). Methylation levels of H3K36 at *FLC* also decreases in *ubp26*; whereas, H3K27me3 levels increase. Thus, these results suggest that UBP26 might regulate *FLC* expression by decreasing the repressive mark H3K27me3 through H2B deubiquitination. Finally, it was shown that the PcG target gene AT1G80160 is also upregulated in *ubp26* mutant (Derkacheva et al., 2016). Taken together, these data show that UBP26 plays an important role in the regulation of the expression of loci located in both heterochromatin and euchromatin.

OTLD1 was found to be interacting with the histone lysine demethylase KDM1C *in planta*. Indeed, OTLD1 has H2B deubiquitination activity *in vitro* and the KDM1C-OTLD1 complex represses gene expression due to H2Bub1 deubiquitination (Krichevsky et al., 2011).

Other interactions and regulation between DUB members and PcG components have not been reported yet and will require further investigation.

## Remarks and Future Perspectives

The characterisation of DUB proteins in different model organisms has accelerated but our understanding of their cellular functions in Arabidopsis remains limited.

In other organisms, DUBs commonly interact physically with partner proteins which determine their protein conformation and target specificity (Larsen et al., 1998; Amerik and Hochstrasser, 2004). AtUBP12 and UBP13 need to interact with the transcription factor MYC2, a regulator of the jasmonate-response pathway, to stabilise MYC2 as a substrate and ultimately affect the JA responses (Jeong et al., 2017), which suggests that protein–protein interactions will also be important in plant DUB function. Similarly, BAP-1 needs to be activated by the DEUBAD domain of ASXL1-3 in order to acquire its H2A deubiquitination activity (Sahtoe et al., 2016). In this review, we have highlighted that in yeast and animals DUBs usually function as part of complexes and these interactions are strictly necessary for correct biological function. However, in plants these complexes remain to be described. Therefore, identification of functional Arabidopsis DUB complexes and the proteins of which they are composed will be paramount to fully understanding DUB-mediated mechanisms.

To study Arabidopsis DUBs a promising strategy can be the use of knock-down and overexpression lines instead of knock-out lines as it has been exemplified in this review. This strategy is especially interesting if we consider that several Arabidopsis DUBs mutants show an embryo-lethal phenotype. In addition, phylogenetic analyses demonstrate that many DUBs might share functional redundancy indicating the difficulty of studying them by forward genetic strategies (**Figures 3**–**6**).

In addition, more research on the DUB family will need to be performed on plant species other than Arabidopsis. So far, the second plant species with the most information on this family is rice, another angiosperm, where the preliminary characterisation of DUBs is emerging. OsUBP6 is involved in rice plant growth and is a member of the putative OsUBP family with another 20 members (Moon et al., 2009). WTG1, which shows high sequence similarity to the human OTUB1 and controls kernel size and shape (Huang et al., 2017). Finally, OsOTUB1, another high sequence similarity to the human OTUB1 is involved in grain yield affecting meristematic activity, seeing that *OsOTUB1* mutants increased grain number and grain weight as well as reducing tiller number per plant (Wang et al., 2017). All these phenotypes increase plant architecture for farming purposes. Considering the results from rice and Arabidopsis and sequence conservation in other plant species (**Figure 1**), we can hypothesise that DUB proteins will have a key impact on the regulation of agronomic traits.

The second part of this review was focused on the role of the ubiquitin levels in the control of gene expression *via* histone tail modifications. As mentioned before, in Drosophila H2A deubiquitination by CALYPSO plays an unexpected repressive role in transcription. In Arabidopsis, OTLD1, involved in H2B deubiquitination, can promote gene activation as well as gene repression (Keren and Citovsky, 2017). These examples indicate that this mark can share functional similarities to other epigenetic mechanisms and the study of multi-epigenetic modifications in certain loci in a specific cell type during development will be the next challenge in the field.

Finally, we believe that there is still much to do regarding the role of DUBs in the modification of different histone variants. So far only the role of the humans USP3 in H2A and γH2AX deubiquitination, BRCC36 in γH2AX deubiquitination as well and the yeast Ubp8 in Cse4 deubiquitination were reported (Shao et al., 2009; Sharma et al., 2014; Canzonetta et al., 2015) and no information comes from plants despite the large number of H2A and H2B variants (Bönisch and Hake, 2012; Molden et al., 2015). Taking into account the essential role of histone variants in control of cell cycle, development and diseases in different organisms, including Arabidopsis (Talbert and Henikoff, 2016), deepening the study of this regulatory link between DUBs and histone variants will add another missing piece to the function of DUBs in the epigenetic regulation of gene expression.

## Author Contributions

EM and SF contributed equally to the design and writing of this article.

## Conflict of Interest Statement

The authors declare that the research was conducted in the absence of any commercial or financial relationships that could be construed as a potential conflict of interest.

## References

[B1] Akutsu M. Dikic I. Bremm A. (2016). Ubiquitin chain diversity at a glance. *J. Cell Sci.* 129 875–880. 10.1242/jcs.183954 26906419

[B2] Allis C. D. Jenuwein T. (2016). The molecular hallmarks of epigenetic control. *Nat. Rev. Genet.* 17 487–500. 10.1038/nrg.2016.59 27346641

[B3] Alonso A. G. D. A. Gutiérrez L. Fritsch C. Papp B. Beuchle D. Müller J. (2007). A genetic screen identifies novel polycomb group genes in drosophila. *Genetics* 176 2099–2108. 10.1534/genetics.107.075739 17717194 PMC1950617

[B4] Amerik A. Y. Hochstrasser M. (2004). Mechanism and function of deubiquitinating enzymes. *Biochim. Biophys. Acta Mol. Cell Res.* 1695 189–207. 10.1016/j.bbamcr.2004.10.003 15571815

[B5] Berger S. L. (2007). The complex language of chromatin regulation during transcription. *Nature* 447 407–412. 10.1038/nature05915 17522673

[B6] Block-Schmidt A. S. Dukowic-Schulze S. Wanieck K. Reidt W. Puchta H. (2011). BRCC36A is epistatic to BRCA1 in DNA crosslink repair and homologous recombination in *Arabidopsis thaliana*. *Nucleic Acids Res.* 39 146–154. 10.1093/nar/gkq722 20817926 PMC3017590

[B7] Bönisch C. Hake S. B. (2012). Histone H2A variants in nucleosomes and chromatin: more or less stable? *Nucleic Acids Res.* 40 10719–10741. 10.1093/nar/gks865 23002134 PMC3510494

[B8] Book A. J. Gladman N. P. Lee S. S. Scalf M. Smith L. M. Vierstra R. D. (2010). Affinity purification of the *Arabidopsis* 26 S proteasome reveals a diverse array of plant proteolytic complexes. *J. Biol. Chem.* 285 25554–25569. 10.1074/jbc.M110.136622 20516081 PMC2919120

[B9] Bratzel F. López-Torrejón G. Koch M. Del Pozo J. C. Calonje M. (2010). Keeping cell identity in *Arabidopsis* requires PRC1 RING-finger homologs that catalyze H2A monoubiquitination. *Curr. Biol.* 20 1853–1859. 10.1016/j.cub.2010.09.046 20933424

[B10] Brazma A. Parkinson H. Sarkans U. Shojatalab M. Vilo J. Abeygunawardena N. (2003). ArrayExpress - a public repository for microarray gene expression data at the EBI. *Nucleic Acids Res.* 31 68–71. 10.1093/nar/gkg091 12519949 PMC165538

[B11] Byeong C. M. Man S. C. Yun H. K. Min C. K. Mi S. C. Chan Y. P. (2005). Arabidopsis ubiquitin-specific protease 6 (AtUBP6) interacts with calmodulin. *FEBS Lett.* 579 3885–3890. 10.1016/j.febslet.2005.05.080 15987637

[B12] Calonje M. (2014). Prc1 marks the difference in plant PcG repression. *Mol. Plant* 7 459–471. 10.1093/mp/sst150 24177684

[B13] Canzonetta C. Vernarecci S. Iuliani M. Marracino C. Belloni C. Ballario P. (2015). SAGA DUB-Ubp8 deubiquitylates centromeric histone variant Cse4. *G3* 6 287–298. 10.1534/g3.115.024877 26613948 PMC4751549

[B14] Cao R. Tsukada Y. Zhang Y. (2005). Role of Bmi-1 and Ring1A in H2A ubiquitylation and Hox gene silencing. *Mol. Cell* 20 845–854. 10.1016/j.molcel.2005.12.002 16359901

[B15] Cao Y. Dai Y. Cui S. Ma L. (2008). Histone H2B monoubiquitination in the chromatin of *FLOWERING LOCUS C* regulates flowering time in *Arabidopsis*. *Plant Cell* 20 2586–2602. 10.1105/tpc.108.062760 18849490 PMC2590739

[B16] Chandler J. S. McArdle B. Callis J. (1997). AtUBP3 and AtUBP4 are two closely related *Arabidopsis thaliana* ubiquitin-specific proteases present in the nucleus. *Mol. Gen. Genet.* 255 302–310. 10.1007/s004380050501 9268021

[B17] Chittock E. C. Latwiel S. Miller T. C. R. Müller C. W. (2017). Molecular architecture of polycomb repressive complexes. *Biochem. Soc. Trans.* 45 193–205. 10.1042/BST20160173 28202673 PMC5310723

[B18] Chung C. H. Baek S. H. (1999). Deubiquitinating enzymes: their diversity and emerging roles. *Biochem. Biophys. Res. Commun.* 266 633–640. 10.1006/bbrc.1999.1880 10603300

[B19] Cope G. A. Suh G. S. Aravind L. Schwarz S. E. Zipursky S. L. Koonin E. V. (2002). Role of predicted metalloprotease motif of Jab1/Csn5 in cleavage of Nedd8 from cul1. *Science* 298 608–611. 10.1126/science.1075901 12183637

[B20] Cui X. Lu F. Li Y. Xue Y. Kang Y. Zhang S. (2013). Ubiquitin-specific proteases UBP12 and UBP13 act in circadian clock and photoperiodic flowering regulation in Arabidopsis. *Plant Physiol.* 162 897–906. 10.1104/pp.112.213009 23645632 PMC3668078

[B21] De Jong R. N. Eiso A. B. Diercks T. Truffault V. Daniëls M. Kaptein R. (2006). Solution structure of the human ubiquitin-specific protease 15 DUSP domain. *J. Biol. Chem.* 281 5026–5031. 10.1074/jbc.M510993200 16298993

[B22] Derkacheva M. Liu S. Figueiredo D. D. Gentry M. Mozgova I. Nanni P. (2016). H2A deubiquitinases UBP12/13 are part of the Arabidopsis polycomb group protein system. *Nat. Plants* 2:16126. 10.1038/nplants.2016.126 27525512

[B23] Doelling J. H. Phillips A. R. Soyler-Ogretim G. Wise J. Chandler J. Callis J. (2007). The ubiquitin-specific protease subfamily UBP3/UBP4 is essential for pollen development and transmission in Arabidopsis. *Plant J.* 145 801–813. 10.1104/pp.106.095323 17905865 PMC2048767

[B24] Doelling J. H. Yan N. Kurepa J. Walker J. Vierstra R. D. (2001). The ubiquitin-specific protease UBP14 is essential for early embryo development in *Arabidopsis thaliana*. *Plant J.* 27 393–405. 10.1046/j.1365-313X.2001.01106.x 11576424

[B25] Dohmann E. M. Kuhnle C. Schwechheimer C. (2005). Loss of the CONSTITUTIVE PHOTOMORPHOGENIC9 signalosome subunit 5 is sufficient to cause the *cop/det/fus* mutant phenotype in Arabidopsis. *Plant Cell* 17 1967–1978. 10.1105/tpc.105.032870 15923347 PMC1167545

[B26] Du L. Li N. Chen L. Xu Y. Li Y. Zhang Y. (2014). The ubiquitin receptor DA1 regulates seed and organ size by modulating the stability of the ubiquitin-specific protease UBP15/SOD2 in *Arabidopsis*. *Plant Cell* 26 665–677. 10.1105/tpc.114.122663 24585836 PMC3967032

[B27] Ewan R. Pangestuti R. Thornber S. Craig A. Carr C. O’Donnell L. (2011). Deubiquitinating enzymes AtUBP12 and AtUBP13 and their tobacco homologue NtUBP12 are negative regulators of plant immunity. *New Phytol.* 191 92–106. 10.1111/j.1469-8137.2011.03672.x 21388379

[B28] Frappier L. Verrijzer C. P. (2011). Gene expression control by protein deubiquitinases. *Curr. Opin. Genet. Dev.* 21 207–213. 10.1016/j.gde.2011.02.005 21411309

[B29] Gardner R. G. Nelson Z. W. Daniel E. Gottschling D. E. (2005). Ubp10 / Dot4p regulates the persistence of ubiquitinated histone H2B: distinct roles in telomeric silencing and general chromatin *Mol. Cell. Biol.* 25 6123–6139. 10.1128/MCB.25.14.6123 PMC1168808 15988024

[B30] Glickman M. H. Rubin D. M. Fried V. A. Finley D. (1998). The regulatory particle of the *Saccharomyces cerevisiae* proteasome. *Mol. Cell. Biol.* 18 3149–3162. 10.1128/MCB.18.6.3149 9584156 PMC108897

[B31] Grimaud C. Bantignies F. Pal-Bhadra M. Ghana P. Bhadra U. Cavalli G. (2006). RNAi components are required for nuclear clustering of polycomb group response elements. *Cell* 124 957–971. 10.1016/j.cell.2006.01.036 16530043

[B32] Gross C. T. McGinnis W. (1996). DEAF-1, a novel protein that binds an essential region in a Deformed response element. *EMBO J.* 15 1961–1970. 8617243 PMC450115

[B33] Gusmaroli G. Feng S. Deng X. W. (2004). The *Arabidopsis* CSN5A and CSN5B subunits are present in distinct COP9 signalosome complexes, and mutations in their JAMM domains exhibit differential dominant negative effects on development. *Plant Cell* 16 2984–3001. 10.1105/tpc.104.025999 15486099 PMC527193

[B34] Haas A. L. Warms J. V. B. Hershkog A. Rose I. A. (1982). Ubiquitin-activating enzyme. Mechanism and role in protein-ubiquitin conjugation. *J. Biol. Chem.* 257 2543–2548. 6277905

[B35] Her J. Soo Lee N. Kim Y. Kim H. (2016). Factors forming the BRCA1-A complex orchestrate BRCA1 recruitment to the sites of DNA damage. *Acta Biochim. Biophys. Sin.* 48 658–664. 10.1093/abbs/gmw047 27325824

[B36] Hershko A. Ciechanover A. (1998). The ubiquitin system. *Annu. Rev. Biochem.* 67 425–479. 10.1146/annurev.biochem.67.1.425 9759494

[B37] Hicke L. (2001). Protein regulation by monoubiquitin. *Nat. Rev. Mol. Cell Biol.* 2 195–201. 10.1038/35056583 11265249

[B38] Huang K. Wang D. Duan P. Zhang B. Xu R. Li N. (2017). *WIDE AND THICK GRAIN 1*, which encodes an otubain-like protease with deubiquitination activity, influences grain size and shape in rice. *Plant J.* 91 849–860. 10.1111/tpj.13613 28621888

[B39] Ishikura S. Weissman A. M. Bonifacino J. S. (2010). Serine residues in the cytosolic tail of the T-cell antigen receptor α-chain mediate ubiquitination and endoplasmic reticulum-associated degradation of the unassembled protein. *J. Biol. Chem.* 285 23916–23924. 10.1074/jbc.M110.127936 20519503 PMC2911338

[B40] Isono E. Katsiarimpa A. Müller I. K. Anzenberger F. Stierhof Y.-D. Geldner N. (2010). The deubiquitinating enzyme AMSH3 is required for intracellular trafficking and vacuole biogenesis in *Arabidopsis thaliana*. *Plant Cell* 22 1826–1837. 10.1105/tpc.110.075952 20543027 PMC2910964

[B41] Jamieson K. Rountree M. R. Lewis Z. A. Stajich J. E. Selker E. U. (2013). Regional control of histone H3 lysine 27 methylation in *Neurospora*. *Proc. Natl. Acad. Sci. U.S.A.* 110 6027–6032. 10.1073/pnas.1303750110 23530226 PMC3625340

[B42] Jeong J. S. Jung C. Seo J. S. Kim J.-K. Chua N.-H. (2017). The deubiquitinating enzymes UBP12 and UBP13 positively regulate MYC2 levels in jasmonate responses. *Plant Cell* 29 1406–1424. 10.1105/tpc.17.00216 28536144 PMC5502463

[B43] Joo H.-Y. Zhai L. Yang C. Nie S. Erdjument-Bromage H. Tempst P. (2007). Regulation of cell cycle progression and gene expression by H2A deubiquitination. *Nature* 449 1068–1072. 10.1038/nature06256 17914355

[B44] Katsiarimpa A. Anzenberger F. Schlager N. Neubert S. Hauser M.-T. Schwechheimer C. (2011). The *Arabidopsis* deubiquitinating enzyme AMSH3 interacts with ESCRT-III subunits and regulates their localization. *Plant Cell* 23 3026–3040. 10.1105/tpc.111.087254 21810997 PMC3180808

[B45] Katsiarimpa A. Kalinowska K. Anzenberger F. Weis C. Ostertag M. Tsutsumi C. (2013). The deubiquitinating enzyme AMSH1 and the ESCRT-III subunit VPS2.1 are required for autophagic degradation in *Arabidopsis*. *Plant Cell* 25 2236–2252. 10.1105/tpc.113.113399 23800962 PMC3723623

[B46] Katsiarimpa A. Muñoz A. Kalinowska K. Uemura T. Rojo E. Isono E. (2014). The ESCRT-III-interacting deubiquitinating enzyme AMSH3 is essential for degradation of ubiquitinated membrane proteins in *Arabidopsis thaliana*. *Plant Cell Physiol.* 55 727–736. 10.1093/pcp/pcu019 24486765

[B47] Keren I. Citovsky V. (2016). The histone deubiquitinase OTLD1 targets euchromatin to regulate plant growth. *Sci. Signal.* 9:ra125. 10.1126/scisignal.aaf6767 27999174 PMC9470540

[B48] Keren I. Citovsky V. (2017). Activation of gene expression by histone deubiquitinase OTLD1. *Epigenetics* 12 584–590. 10.1080/15592294.2017.1348446 28703681 PMC5687338

[B49] Kim J. Hake S. B. Roeder R. G. (2005). The human homolog of yeast BRE1 functions as a transcriptional coactivator through direct activator interactions. *Mol. Cell* 20 759–770. 10.1016/j.molcel.2005.11.012 16337599

[B50] Kim J. Y. Oh J. E. Noh Y. S. Noh B. (2015). Epigenetic control of juvenile-to-adult phase transition by the Arabidopsis SAGA-like complex. *Plant J.* 83 537–545. 10.1111/tpj.12908 26095998

[B51] Komander D. Clague M. J. Urbé S. (2009). Breaking the chains: structure and function of the deubiquitinases. *Nat. Rev. Mol. Cell Biol.* 10 550–563. 10.1038/nrm2731 19626045

[B52] Krichevsky A. Zaltsman A. Lacroix B. Citovsky V. (2011). Involvement of KDM1C histone demethylase-OTLD1 otubain-like histone deubiquitinase complexes in plant gene repression. *Proc. Natl. Acad. Sci. U.S.A.* 108 11157–11162. 10.1073/pnas.1014030108 21690391 PMC3131378

[B53] Kumar S. Stecher G. Tamura K. (2016). MEGA7: molecular evolutionary genetics analysis version 7.0 for bigger datasets. *Mol. Biol. Evol.* 33 1870–1874. 10.1093/molbev/msw054 27004904 PMC8210823

[B54] Kwok S. F. Solano R. Tsuge T. Chamovitz D. A. Ecker J. R. Matsui M. (1998). Arabidopsis homologs of a c-Jun coactivator are present both in monomeric form and in the COP9 complex, and their abundance is differentially affected by the pleiotropic cop / det / fus mutations. *Plant Cell* 10 1779–1790. 10.1105/tpc.10.11.1779 9811788 PMC143959

[B55] Larsen C. N. Krantz B. A. Wilkinson K. D. (1998). Substrate specificity of deubiquitinating enzymes: ubiquitin C-terminal hydrolases. *Biochemistry* 37 3358–3368. 10.1021/bi972274d 9521656

[B56] Li B. Carey M. Workman J. L. (2007). The role of chromatin during transcription. *Cell* 128 707–719. 10.1016/j.cell.2007.01.015 17320508

[B57] Li F. Macfarlan T. Pittman R. N. Chakravarti D. (2002). Ataxin-3 is a histone-binding protein with two independent transcriptional corepressor activities. *J. Biol. Chem.* 277 45004–45012. 10.1074/jbc.M205259200 12297501

[B58] Li W. F. Perry P. J. Prafulla N. N. Schmidt W. (2010). Ubiquitin-specific protease 14 (UBP14) is involved in root responses to phosphate deficiency in *Arabidopsis*. *Mol. Plant* 3 212–223. 10.1093/mp/ssp086 19969521

[B59] Liu Y. Wang F. Zhang H. He H. Ma L. Deng X. W. (2008). Functional characterization of the Arabidopsis *ubiquitin-specific* protease gene family reveals specific role and redundancy of individual members in development. *Plant J.* 55 844–856. 10.1111/j.1365-313X.2008.03557.x 18485060

[B60] Luo M. Luo M. Z. Buzas D. Finnegan J. Helliwell C. Dennis E. S. (2008). Ubiquitin-specific protease 26 is required for seed development and the repression of *PHERES1* in Arabidopsis. *Genetics* 180 229–236. 10.1534/genetics.108.091736 18723879 PMC2535677

[B61] Maertens G. N. El Messaoudi-Aubert S. Elderkin S. Hiom K. Peters G. (2010). Ubiquitin-specific proteases 7 and 11 modulate Polycomb regulation of the *INK4a* tumour suppressor. *EMBO J.* 29 2553–2565. 10.1038/emboj.2010.129 20601937 PMC2928679

[B62] Makarova K. S. Aravind L. Koonin E. V. (2000). A novel superfamily of predicted cysteine proteases from eukaryotes, viruses and *Chlamydia pneumoniae*. *Trends Biochem. Sci.* 25 50–52. 10.1016/S0968-0004(99)01530-3 10664582

[B63] Maytal-Kivity V. Reis N. Hofmann K. Glickman M. H. (2002). MPN+, a putative catalytic motif found in a subset of MPN domain proteins from eukaryotes and prokaryotes, is critical for Rpn11 function. *BMC Biochem.* 3:28. 10.1186/1471-2091-3-28 12370088 PMC129983

[B64] Molden R. C. Bhanu N. V. LeRoy G. Arnaudo A. M. Garcia B. A. (2015). Multi-faceted quantitative proteomics analysis of histone H2B isoforms and their modifications. *Epigenetics Chromatin* 8:15. 10.1186/s13072-015-0006-8 25922622 PMC4411797

[B65] Moon Y. K. Hong J. P. Cho Y. C. Yang S. J. An G. Kim W. T. (2009). Structure and expression of OsUBP6, an ubiquitin-specific protease 6 homolog in rice (*Oryza sativa* L.). *Mol. Cells* 28 463–472. 10.1007/s10059-009-0138-4 19855938

[B66] Moraga F. Aquea F. (2015). Composition of the SAGA complex in plants and its role in controlling gene expression in response to abiotic stresses. *Front. Plant Sci.* 6:865. 10.3389/fpls.2015.00865 26528322 PMC4604261

[B67] Mozgova I. Köhler C. Hennig L. (2015). Keeping the gate closed: functions of the polycomb repressive complex PRC2 in development. *Plant J.* 83 121–132. 10.1111/tpj.12828 25762111

[B68] Mukhopadhyay D. Riezman H. (2007). Proteasome- independent functions of ubiquitin in endocytosis and signaling. *Science* 315 201–205. 10.1126/science.1127085 17218518

[B69] Müller J. Hart C. M. Francis N. J. Vargas M. L. Sengupta A. Wild B. (2002). Histone methyltransferase activity of a *Drosophila* Polycomb group repressor complex. *Cell* 111 197–208. 10.1016/S0092-8674(02)00976-5 12408864

[B70] Nakagawa T. Kajitani T. Togo S. Masuko N. Ohdan H. Hishikawa Y. (2008). Deubiquitylation of histone H2A activates transcriptional initiation via trans-histone cross-talk with H3K4 di- and trimethylation. *Genes Dev.* 22 37–49. 10.1101/gad.1609708 18172164 PMC2151013

[B71] Nicassio F. Corrado N. Vissers J. H. A. Areces L. B. Bergink S. Marteijn J. A. (2007). Human USP3 is a chromatin modifier required for S phase progression and genome stability. *Curr. Biol.* 17 1972–1977. 10.1016/j.cub.2007.10.034 17980597

[B72] Pan R. Kaur N. Hu J. (2014). The Arabidopsis mitochondrial membrane-bound ubiquitin protease UBP27 contributes to mitochondrial morphogenesis. *Plant J.* 78 1047–1059. 10.1111/tpj.12532 24707813

[B73] Pfluger J. Wagner D. (2007). Histone modifications and dynamic regulation of genome accessibility in plants. *Curr. Opin. Plant Biol.* 10 645–652. 10.1016/j.pbi.2007.07.013 17884714 PMC2140274

[B74] Popp M. W. Artavanis-Tsakonas K. Ploegh H. L. (2009). Substrate filtering by the active site crossover loop in UCHL3 revealed by sortagging and gain-of-function mutations. *J. Biol. Chem.* 284 3593–3602. 10.1074/jbc.M807172200 19047059 PMC2635039

[B75] Proost S. Van Bel M. Vaneechoutte D. Van De Peer Y. Inzé D. Mueller-Roeber B. (2015). PLAZA 3.0: an access point for plant comparative genomics. *Nucleic Acids Res.* 43 D974–D981. 10.1093/nar/gku986 25324309 PMC4384038

[B76] Radjacommare R. Usharani R. Kuo C.-H. Fu H. (2014). Distinct phylogenetic relationships and biochemical properties of Arabidopsis ovarian tumor-related deubiquitinases support their functional differentiation. *Front. Plant Sci.* 5:84. 10.3389/fpls.2014.00084 24659992 PMC3950621

[B77] Rando O. J. Ahmad K. (2007). Rules and regulation in the primary structure of chromatin. *Curr. Opin. Cell Biol.* 19 250–256. 10.1016/j.ceb.2007.04.006 17466507

[B78] Robzyk K. Recht J. Osley M. (2000). Rad6-dependent ubiquitination of histone H2B in yeast. *Science* 287 501–504. 10.1126/science.287.5452.501 10642555

[B79] Roudier F. Ahmed I. Bérard C. Sarazin A. Mary-Huard T. Cortijo S. (2011). Integrative epigenomic mapping defines four main chromatin states in Arabidopsis. *EMBO J.* 30 1928–1938. 10.1038/emboj.2011.103 21487388 PMC3098477

[B80] Sahtoe D. D. van Dijk W. J. Ekkebus R. Ovaa H. Sixma T. K. (2016). BAP1/ASXL1 recruitment and activation for H2A deubiquitination. *Nat. Commun.* 7:10292. 10.1038/ncomms10292 26739236 PMC4729829

[B81] Sanchez-Pulido L. Devos D. Sung Z. Calonje M. (2008). RAWUL: a new ubiquitin-like domain in PRC1 Ring finger proteins that unveils putative plant and worm PRC1 orthologs. *BMC Genomics* 9:308. 10.1186/1471-2164-9-308 18588675 PMC2447854

[B82] Sato Y. Yoshikawa A. Yamagata A. Mimura H. Yamashita M. Ookata K. (2008). Structural basis for specific cleavage of Lys 63-linked polyubiquitin chains. *Nature* 455 358–362. 10.1038/nature07254 18758443

[B83] Schatlowski N. Stahl Y. Hohenstatt M. L. Goodrich J. Schubert D. (2010). The CURLY LEAF interacting protein BLISTER controls expression of polycomb-group target genes and cellular differentiation of *Arabidopsis thaliana*. *Plant Cell* 22 2291–2305. 10.1105/tpc.109.073403 20647345 PMC2929108

[B84] Scheuermann J. C. de Ayala Alonso A. G. Oktaba K. Ly-Hartig N. McGinty R. K. Fraterman S. (2010). Histone H2A deubiquitinase activity of the Polycomb repressive complex PR-DUB. *Nature* 465 243–247. 10.1038/nature08966 20436459 PMC3182123

[B85] Scheuermann J. C. Gutiérrez L. Müller J. (2012). Histone H2A monoubiquitination and Polycomb repression: the missing pieces of the puzzle. *Fly* 6 162–168. 10.4161/fly.20986 22836728

[B86] Schmitz R. J. Tamada Y. Doyle M. R. Zhang X. Amasino R. M. (2009). Histone H2B deubiquitination is required for transcriptional activation of *FLOWERING LOCUS C* and for proper control of flowering in Arabidopsis. *Plant Physiol.* 149 1196–1204. 10.1104/pp.108.131508 19091875 PMC2633843

[B87] Shao G. Lilli D. R. Patterson-Fortin J. Coleman K. A. Morrissey D. E. Greenberg R. A. (2009). The Rap80-BRCC36 de-ubiquitinating enzyme complex antagonizes RNF8-Ubc13-dependent ubiquitination events at DNA double strand breaks. *Proc. Natl. Acad. Sci. U.S.A.* 106 3166–3171. 10.1073/pnas.0807485106 19202061 PMC2651241

[B88] Sharma N. Zhu Q. Wani G. He J. Wang Q. E. Wani A. A. (2014). USP3 counteracts RNF168 via deubiquitinating H2A and γH2AX at lysine 13 and 15. *Cell Cycle* 13 106–114. 10.4161/cc.26814 24196443 PMC3925719

[B89] Sievers F. Wilm A. Dineen D. Gibson T. J. Karplus K. Li W. (2014). Fast, scalable generation of high-quality protein multiple sequence alignments using Clustal Omega. *Mol. Syst. Biol.* 7 539–539. 10.1038/msb.2011.75 21988835 PMC3261699

[B90] Sridhar V. V. Kapoor A. Zhang K. Zhu J. Zhou T. Hasegawa P. M. (2007). Control of DNA methylation and heterochromatic silencing by histone H2B deubiquitination. *Nature* 447 735–738. 10.1038/nature05864 17554311

[B91] Steinhauer W. R. Walsh R. C. Kalfayan L. J. Hill C. Carolina N. (1989). Sequence and structure of the *Drosophila melanogaster* ovarian tumor gene and generation of an antibody specific for the ovarian tumor protein. *Microbiology* 9 5726–5732. 10.1128/MCB.9.12.5726 2511440 PMC363746

[B92] Sunnerhagen M. Pursglove S. Fladvad M. (2002). The new MATH: homology suggests shared binding surfaces in meprin tetramers and TRAF trimers. *FEBS Lett.* 530 1–3. 10.1016/S0014-5793(02)03330-6 12387856

[B93] Talbert P. B. Henikoff S. (2016). Histone variants on the move: substrates for chromatin dynamics. *Nat. Rev. Mol. Cell Biol.* 18 115–126. 10.1038/nrm.2016.148 27924075

[B94] Taya S. Yamamoto T. Kanai-Azuma M. Wood S. A. Kaibuchi K. (1999). The deubiquitinating enzyme Fam interacts with and stabilizes beta-catenin. *Genes Cells* 4 757–767. 10.1046/j.1365-2443.1999.00297.x 10620020

[B95] Tian G. Lu Q. Kohalmi S. E. Rothstein S. J. Cui Y. (2012). Evidence that the Arabidopsis ubiquitin C-terminal Hydrolases 1 and 2 associate with the 26S proteasome and the TREX-2 complex. *Plant Signal. Behav.* 7 1415–1419. 10.4161/psb.21899 22951400 PMC3548861

[B96] Van Der Knaap J. A. Kumar B. R. P. Moshkin Y. M. Langenberg K. Krijgsveld J. Heck A. J. R. (2005). GMP synthetase stimulates histone H2B deubiquitylation by the epigenetic silencer USP7. *Mol. Cell* 17 695–707. 10.1016/j.molcel.2005.02.013 15749019

[B97] Verma R. (2002). Role of Rpn11 metalloprotease in deubiquitination and degradation by the 26*S* proteasome. *Science* 298 611–615. 10.1126/science.1075898 12183636

[B98] Vierstra R. D. (2009). The ubiquitin–26S proteasome system at the nexus of plant biology. *Nat. Rev. Mol. Cell Biol.* 10 385–397. 10.1038/nrm2688 19424292

[B99] Wang H. Wang L. Erdjument-Bromage H. Vidal M. Tempst P. Jones R. S. (2004). Role of histone H2A ubiquitination in Polycomb silencing. *Lett. Nat.* 431 873–878. 10.1038/nature02966.1 15386022

[B100] Wang J. Yu Y. Zhang Z. Quan R. Zhang H. Ma L. (2013). *Arabidopsis* CSN5B interacts with VTC1 and modulates ascorbic acid synthesis. *Plant Cell* 25 625–636. 10.1105/tpc.112.106880 23424245 PMC3608782

[B101] Wang S. Wu K. Qian Q. Liu Q. Li Q. Pan Y. (2017). Non-canonical regulation of SPL transcription factors by a human OTUB1-like deubiquitinase defines a new plant type rice associated with higher grain yield. *Cell Res.* 27 1142–1156. 10.1038/cr.2017.98 28776570 PMC5587855

[B102] Weake V. M. Lee K. K. Guelman S. Lin C.-H. Seidel C. Abmayr S. M. (2008). SAGA-mediated H2B deubiquitination controls the development of neuronal connectivity in the *Drosophila* visual system. *EMBO J.* 27 394–405. 10.1038/sj.emboj.7601966 18188155 PMC2234343

[B103] Wolffe A. P. Matzke M. A. (1999). Epigenetics: regulation through repression. *Science* 286 481–486. 10.1126/science.286.5439.481 10521337

[B104] Wood A. Schneider J. Dover J. Johnston M. Shilatifard A. (2003). The Paf1 complex is essential for histone monoubiquitination by the Rad6-Bre1 complex, which signals for histone methylation by COMPASS and Dot1p. *J. Biol. Chem.* 278 34739–34742. 10.1074/jbc.C300269200 12876294

[B105] Wood A. Schneider J. Dover J. Johnston M. Shilatifard A. (2005). The Bur1/Bur2 complex is required for histone H2B monoubiquitination by Rad6/Bre1 and histone methylation by COMPASS. *Mol. Cell* 20 589–599. 10.1016/j.molcel.2005.09.010 16307922

[B106] Worden E. J. Padovani C. Martin A. (2014). Structure of the Rpn11–Rpn8 dimer reveals mechanisms of substrate deubiquitination during proteasomal degradation. *Nat. Struct. Mol. Biol.* 21 220–227. 10.1038/nsmb.2771 24463465

[B107] Xu L. Ménard R. Berr A. Fuchs J. Cognat V. Meyer D. (2009). The E2 ubiquitin-conjugating enzymes, AtUBC1 and AtUBC2, play redundant roles and are involved in activation of *FLC* expression and repression of flowering in *Arabidopsis thaliana*. *Plant J.* 57 279–288. 10.1111/j.1365-313X.2008.03684.x 18798874

[B108] Xu Y. Jin W. Li N. Zhang W. Liu C. Li C. (2016). UBIQUITIN-SPECIFIC PROTEASE 14 interacts with ULTRAVIOLET-B INSENSITIVE 4 to regulate endoreduplication and cell and organ growth in Arabidopsis. *Plant Cell* 28 1200–1214. 10.1105/tpc.16.00007 27099260 PMC4904675

[B109] Yan N. Doelling J. H. Falbel T. G. Durski A. M. Vierstra R. D. (2000). The ubiquitin-specific protease family from Arabidopsis. *At*UBP1 and 2 are required for the resistance to the amino acid analog canavanine. *Plant Physiol.* 124 1828–1843. 10.1104/pp.124.4.1828 11115897 PMC59878

[B110] Yang P. Smalle J. Lee S. Yan N. Emborg T. J. Vierstra R. D. (2007). Ubiquitin C-terminal hydrolases 1 and 2 affect shoot architecture in Arabidopsis. *Plant J.* 51 441–457. 10.1111/j.1365-313X.2007.03154.x 17559514

[B111] Ye H. Park Y. C. Kreishman M. Kieff E. Wu H. (1999). The structural basis for the recognition of diverse receptor sequences by TRAF2. *Mol. Cell* 4 321–330. 10.1016/S1097-2765(00)80334-2 10518213

[B112] Zhang X. Y. Varthi M. Sykes S. M. Phillips C. Warzecha C. Zhu W. (2008). The putative cancer stem cell marker USP22 is a subunit of the human SAGA complex required for activated transcription and cell-cycle progression. *Mol. Cell* 29 102–111. 10.1016/j.molcel.2007.12.015 18206973 PMC2254522

[B113] Zhao J. Zhou H. Zhang M. Gao Y. Li L. Gao Y. (2016). Ubiquitin-specific protease 24 negatively regulates abscisic acid signalling in *Arabidopsis thaliana*. *Plant Cell Environ.* 39 427–440. 10.1111/pce.12628 26290265

[B114] Zheng N. Shabek N. (2017). Ubiquitin ligases: structure, function, and regulation. *Annu. Rev. Biochem.* 86 129–157. 10.1146/annurev-biochem-060815-014922 28375744

[B115] Zhou H. Zhao J. Cai J. Patil S. B. (2017). UBIQUITIN-SPECIFIC PROTEASES function in plant development and stress responses. *Plant Mol. Biol.* 94 565–576. 10.1007/s11103-017-0633-5 28695315

[B116] Zhou H. Zhao J. Yang Y. Chen C. Liu Y. Jin X. (2012). Ubiquitin-specific protease16 modulates salt tolerance in *Arabidopsis* by regulating Na^+^/H^+^ antiport activity and serine hydroxymethyltransferase stability. *Plant Cell* 24 5106–5122. 10.1105/tpc.112.106393 23232097 PMC3556978

[B117] Zhu B. Zheng Y. Pham A. D. Mandal S. S. Erdjument-Bromage H. Tempst P. (2005). Monoubiquitination of human histone H2B: the factors involved and their roles in HOX gene regulation. *Mol. Cell* 20 601–611. 10.1016/j.molcel.2005.09.025 16307923

[B118] Zhu P. Zhou W. Wang J. Puc J. Ohgi K. A. Erdjument-Bromage H. (2007). A histone H2A deubiquitinase complex coordinating histone acetylation and H1 dissociation in transcriptional regulation. *Mol. Cell* 27 609–621. 10.1016/j.molcel.2007.07.024 17707232 PMC2709280

